# Supplementary material for: Patients’ Perception and Knowledge about Influenza and Pneumococcal Vaccination during the COVID-19 Pandemic: An Online Survey in Patients at Risk of Infections
Source: Vaccines (Basel). 2021 Nov 22;9(11):1372. doi: 10.3390/vaccines9111372 (PMC8623007; doi:10.3390/vaccines9111372)
Supplement: Supplementary file 1 [file vaccines-09-01372-s001.zip › vaccines-1476203-supplementary.pdf]

Questionnaire IPSOS

Part 1: Screener

Q0. In the list following, what is the disease or health problem you mainly suffer, for which you are under a doctor's care?
First, the main?
Then, what are the others diseases for which you are under a doctor's care?
Please select several answers.

1. Asplenia
2. Severe asthma needing a treatment
3. Chronic obstructive pulmonary disease (COPD)
4. Cancer (solid tumor or hematologic malignancy treated by chemotherapy)
5. Congenital cardiopathy
6. Hereditary immunodeficiency disorder
7. Diabetes unbalanced by simple diet
8. Sickle cell anemia
9. Emphysema
10. Solid Organ Transplant (heart, lungs, kidney, liver, pancreas)
11. Organ Transplant with donor
12. Hematopoietic stem cell transplanted
13. Pulmonary hypertension (or chronic thromboembolic)
14. HIV infection
15. Chronic heart failure (CHF)
16. Chronic hepatic failure
17. Chronic kidney failure (dialysis)
18. Chronic kidney failure before supplementation
19. Chronic kidney failure (transplanted)
20. Chronic respiratory failure
21. Acute leukemia
22. Chronic myeloid leukemia
23. Lupus
24. Lymphoma
25. Autoimmune diseases treated with immunosuppressive therapy including biologics and/or systemic corticoids
26. Inflammatory bowel diseases
27. Rheumatoid polyarthritis
28. Psoriasis
29. Psoriatic Arthritis
30. Sclerodermia
31. Ankylosing spondylitis
32. Nephrotic syndrome
33. Other autoimmune diseases
34. Neither autoimmune disease
35. Don’t wish to ranswer

Part 2: Identifying information

RS1. You are:
Please select one response.
- Male
- Female

RS2. **How old are you?**
Please rank your response in numbers.
- Under 20
- From 21 to 25
- From 26 to 30
- From 31 to 35
- From 36 to 40
- From 41 to 45
- From 46 to 50
- From 51 to 55
- From 56 to 60
- From 61 to 65
- From 66 to 70
- More than 70

RS3. What is your occupation?
Please select one response.
- Farmers, farm workers
- Freelance
- Senior executives
- Intermediate professions
- Employees
- Workers
- Retired
- Inactives

RS4. Including yourself, how many people currently live in your household?
Please rank the number
_ people

RS5. Which French region do you live in?
Please select one response.
- Auvergne-Rhône-Alpes
- Bourgogne-Franche-Comté
- Bretagne
- Centre-Val de Loire
- Corse
- Grand-Est

- Hauts-de-France
- Ile-de-France
- Normandie
- Nouvelle Aquitaine
- Occitanie
- Pays de la Loire
- Provence-Alpes-Côte d'Azur

- DROM/COM :
  - Martinique
  - Guadeloupe
  - Guyane
  - La Réunion
  - Mayotte
  - Nouvelle-Calédonie
  - Polynésie française
  - Autres DROM/COM

RS6. Are you member of one or several patients associations?
Please select one response.
- Yes
- No

<u>Part3: History with the disease</u>
RS7. Which of the following treatments are you taking for your condition?
Please rank all the responses that apply
- Biotherapy
- Systemic corticotherapy (oral or injection)
- Chemotherapy (oral or injection)
- Anti-rejection drugs
- Other immunosuppressive treatment
- Bronchodilator
- Insulin/Anti-diabetic oral treatment
- Antiretroviral drugs
- Other
- You are not taking any treatment for your disease (exclusive)
- You are not taking any treatment at this moment (exclusive)
- Not wish to respond (exclusive)

RS8. How long have you been taking this treatment?
One answer by type of treatment
- Less than one year
- From 1 to 2 years

- From 3 to 4 years old
- From 5 to 9 years old
- From 10 to 14 years old
- From 15 to 19 years old
- 20 years or more

RS9. Have you in your daily routine one person who provide you care or is regularly present to help you in addition to the healthcare professional (family member, friend, neighbour)?
Please select one response.

- Yes
- No

RS10. How many times per year do you visit a medical doctor?
Only one answer possible per line

- Several times a month
- Once a month
- Once every 2-3 months (about 4 times a year)
- Once or twice a year
- Less often

1. Your attending physician
2. Your specialist doctor who mainly follows you

RS11. Among the listed healthcare professionals, which one is monitoring your vaccinations?
Please select one response

1. A general practitioner
2. A specialist doctor at the hospital
3. A specialist doctor in town
4. A pharmacist
5. A nurse
6. Other

RS12. Do you know where all of your vaccinations are recorded (by you or by your doctor)?
Please select one response

- Yes
- No

1. A health record
2. A vaccination record
3. A Shared Medical Record
4. Other

Part 4: Knowledge and opinions about vaccination

Q1. Would you say you are in favor of vaccination or not?
Please select one response

- Very favorable
- Rather favorable
- Rather not favorable
- Not at all favorable

Q2. Do you know which vaccines are specifically recommended for your illness or treatment?
Please select one response

- Yes
- No

Q3. Do you know about pneumococcal infections?
Please select one response

- Yes and I know quite precisely what it is
- Yes but only in name
- No, not even a name

Q4. For each of the following three infections, would you say that you are more or less likely than the general population to catch them?
Please select only one answer per subject

- More risks than the average of the population
- As many risks as the average of the population
- Less risk than the average of the population

1. The flu
2. Pneumococcal infections
3. Covid-19 (due to SARS-Coronavirus 2)

Q5. For each of the following three diseases, would you say that if you caught them, the consequences would be more or less serious for you than for the average of the general population?
Please select only one answer per subject

- *Much more serious for you*
- *A little more serious for you*
- *Neither more nor less serious for you than for the average of the general population*

1. The flu
2. Pneumococcal infections
3. Covid-19 (due to SARS-Coronavirus 2)

Q6. During the past 12 months, have the listed below healthcare professionals discussed each of these topics with you?
Check all the professionals who have discussed these subjects with you.
Several answers possible per item.

- Your general practitioner
- Your specialist doctor
- A pharmacist
- A nurse
- None of them

1. Recommend that you get the flu vaccine because of your medical condition
2. Make sure you are up to date with your flu vaccine
3. Check that you are up to date with your pneumococcal vaccination (pneumococcal pneumonia)
4. Explain to you that if you get the flu or a pneumococcal infection it could have serious consequences for you.

Q7. In your opinion, at what step of your treatment the doctor should discuss the vaccination?
Please select one response

- As soon as the disease is announced
- When you start your treatments
- When you manage your medication and your illness well

Part 5: Vaccination coverage of patients (influenza and pneumococcal infections)

Part 5a. Influenza vaccines focus

Q8. Have you been offered a flu vaccination during the winter of 2019/2020?
Please select one response

- Yes
- No

Q9. Have you been vaccinated against the flu during winter 2019/2020?
Please select one response

- *Yes*
- *No*
- *I don't know*

Q10. Who did suggest you to get the flu vaccine in winter 2019/2020?
Three possible answers

1. The specialist doctor who follows you
2. Your general practitioner
3. Another doctor
4. A nurse

5. A member of your family
6. A pharmacist
7. An association
8. The health insurance vaccination voucher
9. A campaign
10. No one (exclusive)
11. Other

Q11. Who did perform the flu vaccine in 2019/2020?
Please select one response
1. The specialist doctor who follows you
2. Your general practitioner
3. Another doctor
4. A nurse
5. A pharmacist
6. Yourself
7. Other

Q12. What are the main reasons you were not vaccinated?
Please select two answers
- *In first*
- *In second*

1. We did not offer it to you
2. You doubt the effectiveness of the vaccine
3. You are afraid of the side effects of the vaccine
4. You don't feel at risk for the flu
5. You are against vaccination in general
6. You forgot to get the vaccine
7. You did not have time
8. You think it's a mild illness
9. A health professional advises against it
10. You know that this disease can be treated well
11. Your relatives advise against it
12. For practical reasons (unavailability of the vaccine, find the professional, get an appointment, get the right one, etc.)
13. You do not need to be vaccinated because you are vigilant and adopt barrier gestures
14. Other

<u>Part 5b: Focus vaccines PNEUMOCOCCAL</u>

Q13. Would you say you are up to date with your pneumococcal infection/pneumococcal pneumonia vaccination?
Please select one response
- Yes, I'm sure

- Yes, I think so but I'm not sure
- No, I am not up to date
- I do not know

Q14. The last time you were vaccinated against pneumococcal infections/pneumococcal pneumonia, who did suggest the vaccination?
Please select three answers

1. The specialist doctor who follows you
2. Your general practitioner
3. Another doctor
4. A nurse
5. A member of your family
6. A pharmacist
7. An association
8. A campaign (internet…)
9. Person
10. Other

To those who are vaccinated against pneumococcal infections
Q15. Who did perform the vaccination against pneumococcal infections the last time?
Please select only one answer

1. The specialist doctor who follows you
2. Your general practitioner
3. Another doctor
4. A nurse
5. Other

To those who are not vaccinated against pneumococcal infections
Q16. Why haven't you been vaccinated against pneumococcal infections?
2 responses

- In first
- In second

1. We did not offer it to you
2. You doubt the effectiveness of the vaccine
3. You are afraid of the side effects of the vaccine
4. You don't feel at risk for pneumococcal infections
5. You are against vaccination in general
6. You forgot to get the vaccine
7. For lack of time
8. It is a mild illness
9. A health professional advises against it
10. You know that this disease can be treated well
11. Your relatives advise against it

12. For practical reasons (unavailability of the vaccine, find the professional, get an appointment, etc.)
13. You do not need to be vaccinated because you are vigilant and adopt barrier gestures
14. Other

To those who are vaccinated against pneumococcal infections
Q18. Did you find it easy or not, to understand the vaccine regimen prescribed for you to protect yourself against pneumococcal infections / pneumococcal pneumonia?
Please select only one answer

- Very easy
- Rather easy
- Quite difficult
- Very difficult
- I don't know, I don't know well enough

Q19. Could you be encouraged to get vaccinated (whether against influenza or pneumococcal infections) if you knew that…?
Please select only one answer

- Certainly yes
- Yes, probably
- No, probably not
- No, definitely not

1. Your doctor was himself vaccinated
2. Your immediate circle was vaccinated

<u>Part 6: Optimization track</u>

Q20. For you, is it easy or difficult to get information about ...?
Please select only one answer per item

- Very easy
- Rather easy
- Rather difficult
- Very difficult

1. The vaccines that are recommended because of my state of health or my treatments
2. The best time to get vaccinated
3. The reasons why I need to be vaccinated
4. The diseases against which these vaccines protect
5. Where I can get the vaccines I need
6. Healthcare professionals who can vaccinate me
7. The level of reimbursement of vaccines by Social Security

Q21. Regarding vaccines, would you like more specific information about…?
Only one answer possible
- In 1st
- In 2nd
- In 3rd

1. The vaccines that are recommended because of my state of health or my treatments
2. The best time to get vaccinated
3. The reasons why I need to be vaccinated
4. The diseases against which these vaccines protect
5. Where I can get the vaccines I need
6. Healthcare professionals who can vaccinate me

Q22. What are the three sources of information that you trust the most today to learn about vaccination?
Three possible answers

1. The general practitioner
2. The specialist doctor
3. The pharmacist
4. Public authorities (Ministry of Health, Social Security, etc.)
5. The nurse
6. A patient association
7. Blogs, forums that give advice on what vaccines to do
8. Sites specializing in health (Doctissimo, e-health, etc.)
9. Social networks (Facebook, Twitter, etc.)
10. The media (TV, radio, press)
11. Pharmaceutical laboratories
12. The occupational physician
13. Close entourage
14. The pension fund
15. Supplementary health
16. Other patients with whom you speak

Q23. Could the following steps make you decide to get vaccinated?
Only one answer possible

- *Yes, definitively*
- *Yes, rather*
- *No, rather not*
- *Not at all*

1. Receive alerts by email/phone/letter to remind you to take your vaccines
2. [To be asked of working people] Systematically raise the subject of vaccination during medical visits to occupational medicine

3. Launch a campaign on vaccination (on television, radio, social networks, etc.).
4. Have an easily accessible digital tool that allows you to know if you are up to date with your vaccines (for you and your doctors)
5. That your doctor has vaccines in stock and can vaccine directly
6. Set up a teleconsultation with the doctor or pharmacist to provide you with a point on vaccination
7. To be able to inform you about vaccines within associations of patients who suffer from the same health problems as you.
8. That health insurance send you information on the pneumococcal pneumonia vaccination at the same time as the influenza vaccine voucher
9. That the pharmacist can give these two vaccines (influenza and pneumococcus) that you need.
10. Systematize, as soon as the disease is announced, the delivery of documents related to vaccination in addition to those already provided

Part 7: Coronavirus questions

Q24. Has the Covid-19 pandemic convinced you or, on the contrary, dissuaded you from taking all the vaccines recommended to you?
Only one answer possible

- Totally convinced
- Rather convinced
- Rather dissuaded
- Totally dissuaded
- Neither convinced nor dissuaded

To all
Q25. Would you say after the coronavirus pandemic, you are going to do the following ...?
Only one answer possible

- *Yes, the coronavirus pandemic convinced me that I had to do it*
- *No, the coronavirus pandemic did not convince me to do so*
- *No, because I already did it before*

1. Check that you are up to date in your vaccination record
2. Get the flu vaccination routinely every year
3. Get vaccinated against pneumococcal infections
4. Get vaccinated against other diseases (eg diphtheria, tetanus, polio, hepatitis, whooping cough, etc.) for which you are not up to date
5. Raise the awareness of the importance of getting vaccinated
6. Get vaccinated by a healthcare professional at your home to avoid any outside contact
